# Identification of cannabis use disorder using the Diagnostic and Statistical Manual of Mental Disorders, Fifth Edition (DSM-5) versus DSM-5-proxy measures: Differences by socio-demographic characteristics

**DOI:** 10.1111/add.70411

**Published:** 2026-04-06

**Authors:** Evan L. Eschliman, Erin M. Annunziato, Nicole D. Fitzgerald, Pia M. Mauro

**Affiliations:** 1 Department of Epidemiology, Columbia University Mailman School of Public Health, New York, NY, USA; 2 Center for Pharmacoepidemiology and Treatment Science, Rutgers Institute for Health, Health Care Policy and Aging Research, New Brunswick, NJ, USA; 3 Department of Biostatistics and Epidemiology, Rutgers School of Public Health, Piscataway, NJ, USA

**Keywords:** cannabis use disorder, DSM-5, measurement, National Survey on Drug Use and Health, nationally representative surveys, socio-demographic differences, substance use disorders

## Abstract

**Background and Aims:**

Cannabis use disorder (CUD) is a pressing public health concern in the United States, and understanding trends in prevalence requires considerations of how changes in measurement influence identification of CUD. Starting in 2021, the National Survey on Drug Use and Health (NSDUH) assessed CUD using all 11 *Diagnostic and Statistical Manual of Mental Disorders*, Fifth Edition (DSM-5) criteria in their entire sample; before then, studies could use a nine-criteria DSM-5-proxy measure based on shared DSM-IV criteria. We aimed to identify sociodemographic characteristics associated with additionally identified CUD when using the full DSM-5 measure compared with the DSM-5-proxy measure.

**Design:**

Observational study using nationally representative data.

**Setting:**

United States.

**Participants:**

39 973 participants ages 12 + who reported past-year cannabis use in the 2021–2023 NSDUH (weighted *N* = 57 872 556).

**Measurements:**

Additionally identified mild, moderate, or severe CUD was defined as meeting 2–3, 4–5, or 6 + of the 11 DSM-5 criteria and not meeting the same thresholds with the nine DSM-5-proxy criteria. Multinomial logistic regression models compared characteristics of people with additionally identified mild (vs. none), moderate (vs. mild), and severe (vs. moderate) CUD. We also calculated the percentage of people meeting each of the 11 criteria who had additionally identified CUD.

**Findings:**

Among people who reported past-year cannabis use, 30.5% had DSM-5 CUD, including 7.9% with additionally identified mild CUD not previously identified using the DSM-5-proxy measure, 6.9% with additionally identified moderate CUD and 3.4% with additionally identified severe CUD. People who were younger (vs. 35–49); multiracial (vs. white); non-Hispanic American Indian or Alaska Native (vs. white); or publicly insured or uninsured (vs. privately insured only) were more likely to have additionally identified CUD and/or CUD severity. People who were older (vs. 35–49); Hispanic (vs. white); were non-Hispanic Asian, Native Hawaiian, or Pacific Islander (vs. white); reported female sex (vs. male); or had annual income greater than $75 000 (vs. <$20 000) were less likely to have additionally identified CUD and/or CUD severity. Additionally identified CUD was most common among those meeting “craving” (25.0%), “withdrawal” (20.3%), and “spending time” (14.8%) criteria.

**Conclusions:**

The full *Diagnostic and Statistical Manual of Mental Disorders*, Fifth Edition (DSM-5) measure of cannabis use disorder (CUD) additionally identified CUD differentially across sociodemographic groups in the United States, compared with the DSM-5-proxy measure. Some groups (e.g., people younger than 35 years old, people with any public or no insurance, and people who are multi-racial or non-Hispanic American Indian or Alaska Native) may need more CUD-related services and support than previously expected.

## INTRODUCTION

Cannabis use disorder (CUD) is an ongoing public health concern [1–12]. Epidemiological evidence on trends in CUD in the United States (US) is mixed on whether overall prevalence is increasing [13–15], but relatively consistent on prevalence decreasing among people who have used cannabis longer or more frequently [13–17]. This pattern underscores the importance of considering how changes in measurement based on the Diagnostic and Statistical Manual of Mental Disorders (DSM) diagnostic criteria can influence the identification of CUD, especially mild CUD [15]. The DSM-5, published in 2013, moved away from distinct hierarchical diagnoses of substance ‘dependence’ and ‘abuse’ and adopted a unidimensional definition of substance use disorders, with severity distinguished by symptom count [18–20]. The 11 DSM-5 diagnostic criteria for CUD exclude the DSM-IV criterion of ‘experiencing legal problems’ and include two new criteria of ‘craving’ and ‘withdrawal’ [18].

The US National Survey on Drug Use and Health (NSDUH) aligned its questionnaire items to DSM-5 CUD criteria in 2020 by piloting questions related to ‘craving’ and ‘withdrawal,’ which were administered to the full sample in 2021 [21, 22]. When using NSDUH data from before 2020, several studies estimated DSM-5 CUD using a ‘DSM-5-proxy’ measure, which included the nine DSM-5 CUD criteria shared across the DSM-IV cannabis ‘dependence’ and ‘abuse’ diagnoses [15, 23–32]. Recent studies comparing the full 11-criteria DSM-5 and nine-criteria DSM-5-proxy measures found that almost all moderate and severe cases of DSM-5 CUD were identifiable with either measure, but only 53.2% of people with mild CUD were identifiable with the DSM-5-proxy measure [33, 34]. Although these studies established that the full DSM-5 measure is more inclusive of mild CUD, differential misclassification between the DSM-5 and DSM-5-proxy CUD measures across socio-demographic groups remains underexplored.

We used pooled data from the 2021 to 2023 NSDUH to investigate how additionally identified CUD using the full DSM-5 measure may differ across socio-demographic groups. Although we expected the full DSM-5 measure would identify more CUD and higher CUD severity, this exploratory study had no predefined hypotheses regarding socio-demographic group differences. Findings can help provide much-needed contextualization of epidemiological research on CUD and direction to further investigation of how criteria’s endorsement and interpretation may differ across groups.

## METHOD

### Data source and sample

We used public-use datasets from the 2021 to 2023 NSDUH, a household-based survey of non-institutionalized US residents age 12 years and older that uses multi-stage area probability sampling to estimate a nationally representative sample [22, 35–39].

Of the 41 078 (23.6%) individuals who reported past-year cannabis use, we excluded 1105 (0.6%) whose CUD status could not be defined because of missingness in the items needed to derive individual DSM-5 CUD criteria. Excluded individuals were more likely to be 12 to 26 years old; Hispanic, multi-racial or non-Hispanic Black or African American; in a nonmetropolitan area; and interviewed in 2021 (Tables S1 and S2). The analytic sample included *n* = 39 973 individuals (weighted *n* = 57 872 556).

### Measures

#### Cannabis use disorder

Individuals were classified as having DSM-5 CUD if they met two or more of the 11 DSM-5 criteria and DSM-5-proxy CUD if they met two or more of the nine DSM-IV criteria (all DSM-5 criteria except ‘craving’ and ‘withdrawal’ and excluding the DSM-IV criterion of ‘legal problems’). CUD severity was based on number of criteria met: mild (2–3), moderate (4–5) or severe (6–11 for DSM-5, 6–9 for DSM-5-proxy) [18]. Table S3 summarizes NSDUH items used to determine DSM-5 and DSM-5-proxy CUD criteria.

We, then, created a categorical variable with three mutually exclusive categories: (1) no CUD using both measures; (2) CUD using both measures; or (3) DSM-5 CUD, but not DSM-5-proxy CUD (i.e. ‘additionally identified’ CUD, all mild). We also identified participants with a higher past-year CUD severity using the full DSM-5 measure versus the DSM-5-proxy measure.

#### Socio-demographic characteristics

We included the following socio-demographic characteristics previously associated with disparities in CUD and/or CUD treatment [1, 23, 28, 31, 32, 40–42]: age (12–17, 18–25, 26–34, 35–49, 50–64 or ≥65), race/ethnicity (non-Hispanic Asian, Native Hawaiian or Pacific Islander; non-Hispanic Black or African American; Hispanic; non-Hispanic American Indian or Alaska Native; multi-racial; or non-Hispanic White), reported binary sex (female or male), insurance status (only private insurance, any public insurance, other insurance or uninsured), annual income (<$20 000, $20 000–49 999, $50 000–74 999 or ≥$75 000), urbanicity (large metropolitan area, small metropolitan area or non-metropolitan area), and survey year (2021, 2022 or 2023).

### Statistical analysis

All analyses used *svy* procedures in Stata version 19 to account for complex survey methodology [43]. We first estimated sample characteristics of people reporting past-year cannabis use along with the prevalence of past-year DSM-5 CUD and additionally identified CUD severity. We used multinomial logistic regression models to estimate the unadjusted and adjusted relative risk ratios (RRR; aRRR) and corresponding 95% CI of having: (1) additionally identified mild CUD (reference: no CUD using both measures); (2) additionally identified moderate CUD (reference: mild CUD using both measures); and (3) additionally identified severe CUD (reference: moderate CUD using both measures). We used the largest subgroup as the reference group for each socio-demographic variable except for income, which used the reference of less than $20 000 for interpretability. In sensitivity analyses, we adjusted for past-year daily or near-daily cannabis use frequency (≥300 days).

Last, we estimated percentages of people who reported past-year cannabis use meeting each criterion, and, among those meeting each criterion, the percentage having additionally identified CUD. In *ad hoc* analyses, we estimated relative risks (RR) of meeting the additional criteria (i.e. ‘craving’ and ‘withdrawal’). In this exploratory study, model results were considered statistically significant if the 95% CI did not include the null [44, 45]. No ethical approval was required for this secondary analysis of publicly available de-identified survey data.

## RESULTS

### CUD case and severity classification

An estimated 30.5% of people reporting past-year cannabis use had past-year DSM-5 CUD (Table 1). Additionally identified mild CUD comprised 25.8% of all DSM-5 cases, and the DSM-5-proxy measure identified lower severity in 45.3% of cases it identified. An estimated 18.2% of people reporting past-year cannabis use had disagreement between measures: 7.9% had additionally identified mild CUD, 6.9% had additionally identified moderate CUD and 3.4% had additionally identified severe CUD.

### Socio-demographic differences in additionally identified CUD severity

As shown in Table 2, people with additionally identified mild CUD were more likely to be multi-racial versus non-Hispanic white (aRRR = 1.45; 95% CI = 1.07–1.96), have any public insurance versus private only (aRRR = 1.58; 95% CI = 1.35–1.85) and be uninsured versus private only (aRRR = 1.32; 95% CI = 1.10–1.58) compared to people with no CUD using both measures. People with additionally identified mild CUD were less likely to be 65 years old and older versus 35 to 49 (aRRR = 0.20; 95% CI = 0.14–0.28); non-Hispanic Asian, Native Hawaiian or Pacific Islander versus non-Hispanic white (aRRR = 0.53; 95% CI = 0.33–0.83); Hispanic versus non-Hispanic white (aRRR = 0.81; 95% CI = 0.67–0.98); report female versus male sex (aRRR = 0.80; 95% CI = 0.71–0.89); and have an annual income of $75 000 or more versus less than $20 000 (aRRR = 0.77; 95% CI = 0.77–0.96) compared to people with no CUD using both measures.

Additionally identified moderate CUD was more likely among people 18 to 25 years old (aRRR = 1.52; 95% CI = 1.22–1.90) and 26 to 34 years old (aRRR = 1.44; 95% CI = 1.17–1.76) and less likely among 50 to 64 years old (aRRR = 0.55; 95% CI = 0.32–0.95), all versus 35 to 49 years old, compared to mild CUD using both measures. People who had additionally identified severe CUD were more likely to be non-Hispanic American Indian or Alaska Native versus non-Hispanic white (aRRR = 2.76; 95% CI = 1.06–7.21) and have any public insurance versus private only (aRRR = 1.61; 95% CI = 1.05–2.48) and less likely to be 65 years old or older versus 35 to 49 (aRRR = 0.21; 95% CI = 0.05–0.89) compared to people with moderate CUD using both measures.

Sensitivity analyses adjusting for past-year near-daily or daily cannabis use were consistent with the main findings. Moreover, people ages 12 to 35 years were more likely to have additionally identified CUD, and the associations were no longer statistically significant for people identified as Hispanic, uninsured or with annual incomes of $75 000 or more (Table S4).

### CUD criteria

When examining additionally identified CUD among people meeting individual DSM-5 CUD criteria, relatively large percentageswere observed among those who met ‘craving’ (25.0%; 95% CI = 23.5–26.5), ‘withdrawal’ (20.3%; 95% CI = 18.2–22.7) and ‘spending time’ (14.8%; 95% CI = 13.8–15.9) (Figure 1, Table S5). Associations between socio-demographic groups and additionally identified CUD cases or severity were consistent with associations with meeting ‘craving’ or ‘withdrawal’ criteria (Table S6).

## DISCUSSION

One in four CUD cases identified using the full DSM-5 measure were not identified using the DSM-5-proxy measure, which is consistent with prior studies [33, 34]. The DSM-5-proxy measure identified lower CUD severity in approximately 45% of cases, with nearly 23% of additionally identified DSM-5 CUD cases classified as moderate and 11% as severe.

We found evidence for differential misclassification across some socio-demographic groups, particularly related to race/ethnicity, age and insurance. Of particular concern are groups for which the DSM-5-proxy measure identified relatively fewer CUD cases (i.e. multi-racial people, people reporting any public insurance and people who are uninsured) or relatively lower CUD severity (i.e. ages 12–35, non-Hispanic American Indian or Alaska Native people and people reporting any public insurance). Meanwhile, some groups were relatively less likely to have additionally identified CUD (i.e. people who are ≥65 years old; are non-Hispanic Asian, Native Hawaiian or Pacific Islander; report female sex; and have an annual income of ≥$75 000), meaning prior DSM-5-proxy estimates are relatively more similar to those using the full DSM-5 measure. For these groups, measuring ‘craving’ and/or ‘withdrawal’ criteria is particularly important in CUD case definition. Future research can further examine the many social factors related to these socio-demographic characteristics that may influence prevalence and salience of these criteria as well as item interpretation [46–54]. For example, structural racism can pattern the availability of cannabis products [55, 56], which could in turn influence the prevalence of some CUD criteria (e.g. ‘spending time’).

When examining individual criteria, it was expected that people meeting the two criteria unique to the DSM-5 measure (i.e. ‘craving’ and ‘withdrawal’) would have the largest percentages of additionally identified CUD. We unexpectedly saw that nearly 15% of people meeting the ‘spending time’ criterion had additionally identified CUD. This could be partly explained by the frequent co-occurrence of ‘craving’ and ‘spending time’ [46, 47], as well as many of the same underexplored social factors that may underpin the other associations found in this study. The apparent importance of the ‘craving’, ‘withdrawal’ and ‘spending time’ criteria in determining DSM-5 CUD further motivate their potential as targets for prevention and treatment [8, 57].

Our findings identify possibly under-served and under-diagnosed socio-demographic groups who may benefit from greater CUD-related services and supports. CUD treatment use among people with CUD is rare and the prevalence of treatment use appears to have decreased over time [23, 32, 40]. Multi-racial people and non-Hispanic American Indian and Alaska Native people are two groups that face documented disparities in CUD [28, 32, 41] and may have greater treatment need than previously estimated. Adolescent and younger adult age groups (i.e. 12–34 years old) were more likely to have additionally identified moderate CUD as well as additionally identified mild CUD when adjusting for cannabis use frequency. Therefore, these age groups may be an especially important group for tailored prevention, screening, and other forms of early intervention to prevent development of more severe CUD [10, 11, 58, 59].

This study has limitations. We were unable to examine differences in classification compared to the full set of DSM-IV criteria because the ‘legal problems’ DSM-IV criterion was not assessed in the 2021 to 2023 NSDUH. NSDUH has published on the comparability between the full DSM-IV and DSM-5 CUD measures [21]. Notably, NSDUH items’ wording and use for determining CUD may differ from formal diagnostic tools or clinical assessments [18]. Additionally, some socio-demographic groups (e.g. American Indian and Alaska Native people [60]) had relatively small sample sizes and higher missingness, which could limit the precision of these estimates.

## CONCLUSION

Differences in identification of CUD between the DSM-5-proxy and full DSM-5 measures differentially occurred across age, race/ethnicity, sex, insurance status and income. CUD-related needs may have been underestimated among people 12 to 34 years old, with any public or no insurance and people who are multi-racial or non-Hispanic American Indian or Alaska Native. These findings help contextualize research on the epidemiology and nosology of CUD, as well as inform the provision of CUD-related services and supports to groups whose CUD severity may have been underestimated in past research.

## Supplementary Material

Supplemental material

Additional supporting information can be found online in the Supporting Information section at the end of this article.

## Figures and Tables

**FIGURE 1:**
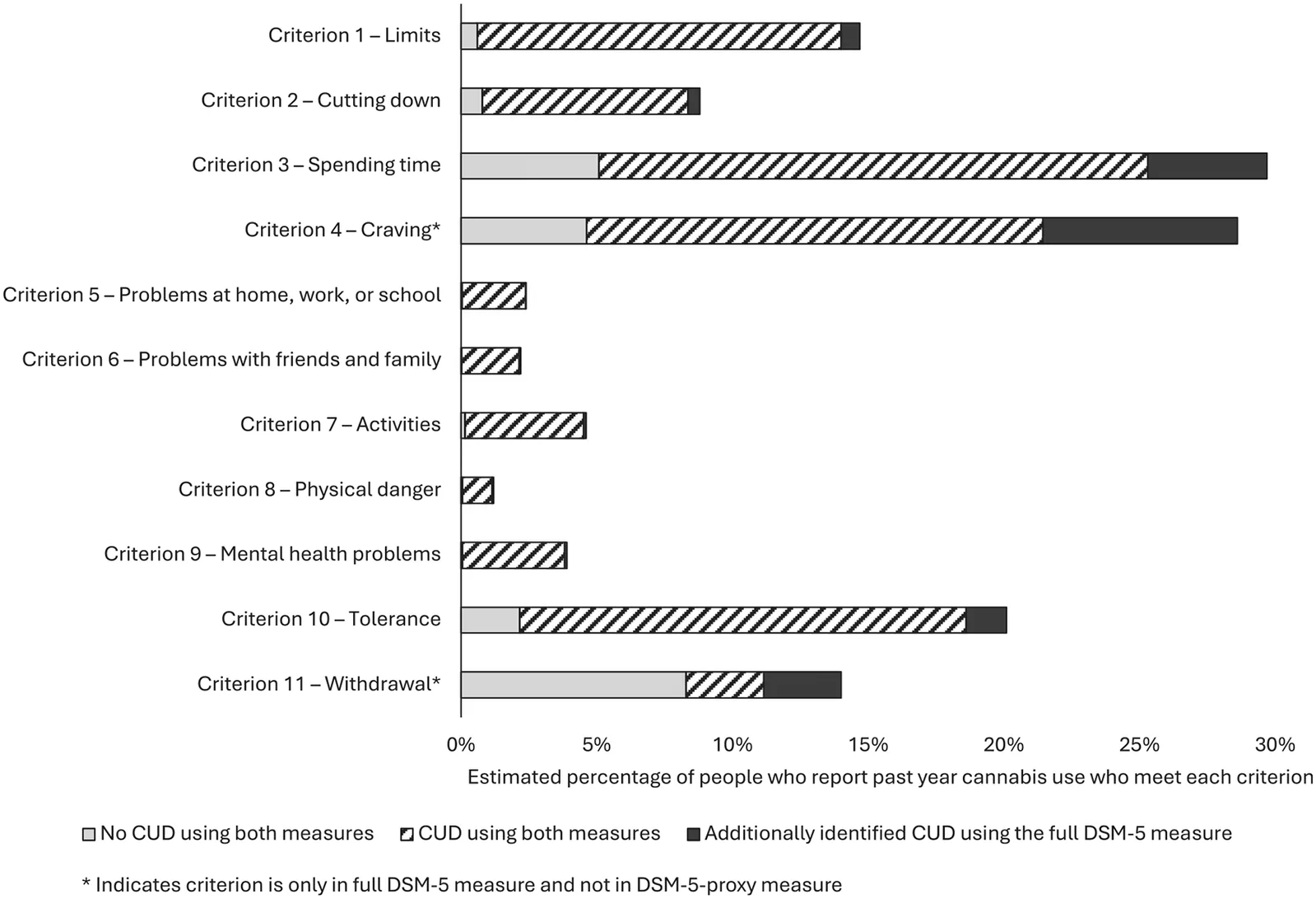
Cannabis use disorder case classification among people who met each individual criterion.

**TABLE 1:** Socio-demographic characteristics of people who report past-year cannabis use and cannabis use disorder case and severity classification.

	Overall sample: past-year cannabis use		DSM-5 CUD	Additionally identified CUD and CUD severity using the full DSM-5 measure		
Socio-demographic variable	Weighted *n*	Column Wt.% (95% CI)	Row Wt.% (95% CI)	Additionally identified mild CUD^a^ Row Wt.% (95% CI)	Additionally identified moderate CUD Row Wt.% (95% CI)	Additionally identified severe CUD Row Wt.% (95% CI)
Total	57 872 556	100.0	30.5 (29.7–31.4)	7.9 (7.4–8.4)	6.9 (6.6–7.3)	3.4 (3.0–3.7)
Age, years						
12–17	2 661 952	4.6 (4.3–4.8)	43.8 (41.2–46.5)	7.7 (6.1–9.7)	9.9 (8.5–11.4)	7.9 (6.5–9.6)
18–25	12 330 142	21.3 (20.6–22.1)	42.6 (41.3–44.0)	7.5 (6.9–8.2)	11.0 (10.2–11.9)	6.5 (5.9–7.2)
26–34	13 391 483	23.1 (22.4–23.9)	34.3 (33.0–35.7)	9.0 (8.1–10.1)	8.9 (8.1–9.9)	3.6 (2.9–4.5)
35–49	14 626 080	25.3 (24.4–26.2)	27.6 (25.7–29.5)	8.6 (7.6–9.8)	5.8 (5.1–6.5)	2.0 (1.5–2.5)
50–64	10 257 581	17.7 (16.8–18.7)	20.7 (17.9–23.7)	8.0 (6.4–9.9)	2.7 (1.9–3.8)	1.4 (0.8–2.3)
≥65	4 605 318	8.0 (7.3–8.7)	11.0 (9.0–13.5)	2.9 (2.1–3.9)	1.7 (0.7–3.7)	0.4 (0.1–1.2)
Race/ethnicity						
Hispanic	9 055 408	15.7 (14.8–16.5)	34.4 (32.4–36.3)	7.0 (6.1–8.0)	8.2 (7.1–9.3)	3.9 (3.3–4.7)
Multi-racial	1 816 998	3.1 (2.9–3.5)	40.2 (35.7–44.9)	11.0 (8.6–13.9)	11.1 (8.6–14.2)	3.6 (2.5–5.2)
NH American Indian or Alaska Native	481 891	0.9 (0.7–1.0)	42.6 (35.4–50.2)	8.7 (5.1–14.3)	11.9 (8.5–16.5)	6.1 (3.7–10.0)
NH Asian, Native Hawaiian or Pacific Islander	1 940 028	3.4 (3.0–3.7)	28.4 (23.5–33.9)	4.2 (2.7–6.5)	6.2 (4.0–9.5)	3.6 (2.4–5.2)
NH Black or African American	7 642 280	13.2 (12.5–13.9)	34.6 (32.7–36.4)	8.7 (7.5–10.1)	7.3 (6.3–8.5)	4.8 (3.9–6.0)
NH White	36 935 951	63.8 (62.6–65.1)	28.3 (27.1–29.4)	7.9 (7.3–8.6)	6.3 (5.9–6.8)	2.9 (2.5–3.3)
Sex						
Male	31 355 935	54.2 (53.2–55.1)	33.4 (32.2–34.6)	8.1 (7.5–8.9)	7.6 (6.9–8.2)	3.7 (3.2–4.2)
Female	26 516 621	45.8 (44.9–46.8)	27.2 (26.2–28.3)	7.5 (6.9–8.2)	6.2 (5.7–6.8)	3.0 (2.6–3.5)
Insurance status						
Private only	28 590 381	49.4 (48.5–50.3)	26.8 (25.7–27.8)	6.9 (6.3–7.5)	5.7 (5.1–6.2)	2.9 (2.6–3.4)
Any public	21 242 340	36.7 (35.8–37.7)	32.8 (31.5–34.1)	8.8 (8.0–9.7)	7.6 (6.7–8.6)	3.7 (3.2–4.2)
Other insurance	1 606 601	2.8 (2.5–3.1)	35.5 (31.3–40.0)	7.9 (5.5–11.3)	7.9 (5.9–10.6)	4.8 (3.1–7.3)
Uninsured	6 433 234	11.1 (10.6–11.7)	38.7 (35.9–41.5)	9.1 (8.0–10.4)	10.2 (8.6–12.0)	3.8 (2.8–5.1)
Annual income						
<$20 000	10 372 934	17.9 (17.0–18.9)	36.0 (34.1–37.9)	9.0 (8.0–10.2)	8.8 (7.9–9.8)	4.2 (3.6–5.0)
$20 000–49 999	16 031 225	27.7 (26.8–28.6)	35.2 (33.4–37.0)	9.2 (8.1–10.5)	7.9 (7.1–8.9)	3.7 (3.1–4.3)
$50 000–74 999	8 487 985	14.7 (13.9–15.4)	32.3 (29.7–35.0)	8.1 (6.9–9.5)	7.2 (6.1–8.4)	3.6 (2.8–4.7)
≥$75 000	22 980 412	39.7 (38.5–40.9)	24.2 (23.0–25.5)	6.3 (5.7–6.9)	5.3 (4.7–6.0)	2.6 (2.3–3.1)
Residence						
Large metropolitan area	32 556 292	56.3 (55.0–57.5)	29.3 (27.9–30.7)	7.2 (6.4–8.1)	6.6 (6.0–7.2)	3.5 (3.1–3.9)
Small metropolitan area	18 448 204	31.9 (30.6–33.2)	31.9 (30.6–33.2)	8.9 (8.2–9.6)	7.0 (6.2–7.9)	3.4 (2.9–4.0)
Non-metropolitan area	6 868 061	11.9 (10.9–12.9)	33.0 (30.5–35.6)	8.2 (7.0–9.5)	8.4 (7.1–9.8)	2.8 (2.3–3.4)
Survey year						
2021	17 195 671	29.7 (28.5–30.9)	30.3 (28.5–32.1)	7.9 (6.8–9.2)	6.9 (6.0–8.0)	3.2 (2.7–3.8)
2022	20 281 161	35.0 (34.0–36.1)	30.7 (29.4–32.1)	7.9 (7.1–8.8)	7.4 (6.6–8.2)	3.4 (3.0–3.8)
2023	20 395 724	35.2 (33.8–36.8)	30.6 (29.3–31.9)	7.8 (7.1–8.5)	6.5 (5.9–7.1)	3.5 (3.0–4.0)

*Note*: Unweighted total sample of *n* = 39 973. Data from the 2021–2023 National Survey on Drug Use and Health.

Abbreviations: CI, confidence interval; CUD, cannabis use disorder; DSM-5, Diagnostic and Statistical Manual of Mental Disorders, Fifth Edition; NH, non-Hispanic; Wt., weighted.

a All instances of additionally identified CUD using the full DSM-5 measure were identified as mild CUD; this group is equivalent to ‘additionally identified CUD using the full DSM-5 measure’.

**TABLE 2:** Correlates of having additionally identified CUD and CUD severity using the full DSM-5 measure among people who report past-year cannabis use.

	Additionally identified mild CUD using the full DSM-5 measure^a^ vs. no CUD using both measures		Additionally identified moderate CUD using the full DSM-5 measure vs. mild CUD using both measures		Additionally identified severe CUD using the full DSM-5 measure vs. moderate CUD using both measures	
Socio-demographic variable	RRR^b^ (95% CI)	aRRR^b^ (95% CI)	RRR^c^ (95% CI)	aRRR^c^ (95% CI)	RRR^c^ (95% CI)	aRRR^c^ (95% CI)
Age, years						
12–17	1.15 (0.92–1.44)	1.15 (0.92–1.45)	**1.40 (1.01–1.95)**	1.39 (1.00–1.93)	1.51 (0.80–2.83)	1.39 (0.76–2.53)
18–25	1.11 (0.92–1.34)	1.11 (0.91–1.34)	**1.50 (1.20–1.86)**	**1.52 (1.22–1.90)**	1.61 (0.98–2.64)	1.65 (0.99–2.75)
26–34	1.16 (0.96–1.39)	1.16 (0.97–1.39)	**1.41 (1.15–1.73)**	**1.44 (1.17–1.76)**	1.69 (0.97–2.94)	1.71 (0.96–3.04)
35–49	1 [Reference]	1 [Reference]	1 [Reference]	1 [Reference]	1 [Reference]	1 [Reference]
50–64	0.85 (0.63–1.16)	0.80 (0.59–1.09)	**0.57 (0.33–1.00)**	**0.55 (0.32–0.95)**	1.02 (0.37–2.80)	1.01 (0.37–2.77)
≥65	**0.27 (0.19–0.38)**	**0.20 (0.14–0.28)**	0.48 (0.15–1.52)	0.42 (0.13–1.34)	0.27 (0.07–1.06)	**0.21 (0.05–0.89)**
Race/ethnicity						
Hispanic	0.96 (0.79–1.16)	**0.81 (0.67–0.98)**	1.03 (0.80–1.32)	0.87 (0.68–1.13)	1.05 (0.65–1.67)	0.87 (0.53–1.41)
Multi-racial	**1.66 (1.24–2.23)**	**1.45 (1.07–1.96)**	1.43 (0.93–2.20)	1.29 (0.85–1.96)	0.82 (0.38–1.79)	0.70 (0.32–1.52)
NH American Indian or Alaska Native	1.37 (0.77–2.43)	1.09 (0.60–1.96)	1.18 (0.78–1.80)	1.03 (0.69–1.52)	**2.88 (1.09–7.66)**	**2.76 (1.06–7.21)**
NH Asian, Native Hawaiian or Pacific Islander	**0.53 (0.33–0.84)**	**0.53 (0.33–0.83)**	0.81 (0.41–1.60)	0.74 (0.37–1.46)	1.32 (0.65–2.65)	1.18 (0.58–2.43)
NH Black or African American	**1.20 (1.01–1.43)**	1.02 (0.84–1.23)	1.01 (0.80–1.26)	0.89 (0.71–1.13)	0.99 (0.58–1.69)	0.84 (0.48–1.50)
NH White	1 [Reference]	1 [Reference]	1 [Reference]	1 [Reference]	1 [Reference]	1 [Reference]
Sex						
Male	1 [Reference]	1 [Reference]	1 [Reference]	1 [Reference]	1 [Reference]	1 [Reference]
Female	**0.85 (0.75–0.96)**	**0.80 (0.71–0.89)**	1.09 (0.89–1.34)	1.04 (0.85–1.27)	1.38 (0.96–1.99)	1.34 (0.94–1.91)
Insurance status						
Private only	1 [Reference]	1 [Reference]	1 [Reference]	1 [Reference]	1 [Reference]	1 [Reference]
Any public	**1.40 (1.23–1.60)**	**1.58 (1.35–1.85)**	1.19 (0.93–1.53)	1.34 (0.98–1.82)	1.30 (0.86–1.95)	**1.61 (1.05–2.48)**
Other insurance	1.31 (0.88–1.95)	1.23 (0.81–1.87)	1.24 (0.79–1.95)	1.23 (0.78–1.93)	0.99 (0.38–2.56)	1.01 (0.39–2.61)
Uninsured	**1.59 (1.36–1.86)**	**1.32 (1.10–1.58)**	1.24 (0.93–1.65)	1.23 (0.90–1.67)	1.30 (0.73–2.32)	1.42 (0.80–2.52)
Annual income						
<$20 000	1 [Reference]	1 [Reference]	1 [Reference]	1 [Reference]	1 [Reference]	1 [Reference]
$20 000–49 999	1.01 (0.84–1.21)	1.11 (0.92–1.33)	0.80 (0.62–1.02)	0.81 (0.63–1.03)	1.13 (0.70–1.84)	1.20 (0.75–1.92)
$50 000–74 999	0.85 (0.69–1.03)	0.99 (0.80–1.23)	0.78 (0.60–1.02)	0.81 (0.61–1.07)	0.81 (0.45–1.44)	0.91 (0.50–1.63)
≥$75 000	**0.59 (0.49–0.71)**	**0.77 (0.62–0.96)**	**0.79 (0.64–0.97)**	0.91 (0.70–1.17)	0.98 (0.59–1.60)	1.23 (0.76–2.00)
Residence						
Large metropolitan area	1 [Reference]	1 [Reference]	1 [Reference]	1 [Reference]	1 [Reference]	1 [Reference]
Small metropolitan area	**1.28 (1.09–1.51)**	1.18 (0.99–1.40)	0.98 (0.79–1.22)	0.94 (0.75–1.19)	1.09 (0.70–1.71)	1.06 (0.68–1.65)
Non-metropolitan area	1.20 (0.97–1.47)	1.06 (0.85–1.33)	1.08 (0.80–1.45)	1.04 (0.77–1.40)	0.68 (0.38–1.21)	0.64 (0.35–1.16)
Survey year						
2021	1 [Reference]	1 [Reference]	1 [Reference]	1 [Reference]	1 [Reference]	1 [Reference]
2022	1.00 (0.82–1.22)	1.03 (0.85–1.24)	1.14 (0.90–1.45)	1.17 (0.92–1.47)	0.96 (0.61–1.50)	0.97 (0.61–1.52)
2023	0.98 (0.81–1.19)	1.04 (0.86–1.26)	0.96 (0.75–1.24)	1.00 (0.77–1.28)	0.79 (0.49–1.28)	0.82 (0.51–1.32)

*Note*: Bolded values indicate 95% CI does not include 1. Data from the 2021–2023 National Survey on Drug Use and Health.

Abbreviations: aRRR, adjusted relative risk ratio; CI, confidence interval; CUD, cannabis use disorder; DSM-5, Diagnostic and Statistical Manual of Mental Disorders, Fifth Edition; NH, non-Hispanic; RRR, relative risk ratio.

a All instances of additionally identified CUD using the full DSM-5 measure were identified as mild CUD; this group is equivalent to ‘additionally identified CUD using the full DSM-5 measure’.

b From a multinomial logistic model with categories of: no CUD using both measures, additionally identified CUD using the full DSM-5 measure and CUD using both measures.

c From multinomial logistic models with categories of: no CUD using both measures; additionally identified mild CUD using the full DSM-5; mild CUD using both measures; additionally identified moderate CUD using the full DSM-5 measure; moderate CUD using both measures; additionally identified severe CUD using the full DSM-5 measure; and severe CUD using both measures.

## Data Availability

The data that support the findings of this study are available in National Survey on Drug Use and Health Data Files at https://www.samhsa.gov/data/data-we-collect/nsduh-national-survey-drug-use-and-health/. These data were derived from the following resources available in the public domain: - 2021 NSDUH Public Data File, https://www.samhsa.gov/data/data-we-collect/nsduh-national-survey-drug-use-and-health/datafiles/2021 - 2022 NSDUH Public Data File, https://www.samhsa.gov/data/data-we-collect/nsduh-national-survey-drug-use-and-health/datafiles/2022 - 2023 NSDUH Public Data File, https://www.samhsa.gov/data/data-we-collect/nsduh-national-survey-drug-use-and-health/datafiles/2023.
