# Supplementary material for: Identification of cannabis use disorder using the Diagnostic and Statistical Manual of Mental Disorders, Fifth Edition (DSM-5) versus DSM-5-proxy measures: Differences by socio-demographic characteristics
Source: Addiction. Author manuscript; Available in PMC 2026 Oct 27. (PMC13712168; doi:10.1111/add.70411)
Supplement: Supplementary file 1 [file NIHMS2211882-supplement-Supplemental_material.docx]

**Supplementary material for *Identification of cannabis use disorder using the DSM-5 versus DSM-5-proxy measures: Differences by sociodemographic characteristics***

**Supplementary Table 1.** Counts of missingness and weighted percentages for each DSM-5 cannabis use disorder criteria

| **DSM-5 CUD Criteria** | **Missing criterion out of all participants**  **(unweighted *N*=173,808)**  n (%) | **Missing criterion out of participants who reported past-year cannabis use**  **(unweighted *N*=41,078)**  n (%) | **Missing criterion out of people reporting past-year cannabis use**  **(weighted *N*=59,057,510)**  Row Wt. % (95% CI) |
| --- | --- | --- | --- |
| Criterion 1 – Limits | 1,239 (0.71%) | 993 (2.42%) | 1.8 (1.6, 2.1) |
| Criterion 2 – Cutting down | 1,257 (0.72%) | 1,011 (2.46%) | 1.8 (1.6, 2.1) |
| Criterion 3 – Spending time | 1,215 (0.70%) | 969 (2.36%) | 1.7 (1.5, 2.0) |
| Criterion 4 – Craving | 1,225 (0.70%) | 979 (2.38%) | 1.8 (1.5, 2.0) |
| Criterion 5 – Problems at home, work, or school | 1,275 (0.73%) | 1,029 (2.50%) | 1.9 (1.6, 2.2) |
| Criterion 6 – Problems with friends and family | 1,287 (0.74%) | 1,041 (2.53%) | 1.9 (1.7, 2.2) |
| Criterion 7 – Activities | 1,274 (0.73%) | 1,028 (2.50%) | 1.9 (1.7, 2.2) |
| Criterion 8 – Physical danger | 1,275 (0.73%) | 1,029 (2.50%) | 1.9 (1.6, 2.2) |
| Criterion 9 – Mental health problems | 1,260 (0.72%) | 1,014 (2.47%) | 1.8 (1.6, 2.1) |
| Criterion 10 – Tolerance | 1,236 (0.71%) | 990 (2.41%) | 1.8 (1.6, 2.1) |
| Criterion 11 – Withdrawal | 1,315 (0.76%) | 1,069 (2.60%) | 1.9 (1.7, 2.2) |

*Notes.* Data from the 2021–2023 National Survey on Drug Use and Health. Because of the rules used to determine missingness of DSM-5 CUD (see Supplemental Table 3), the total number of individuals missing DSM-5 CUD and thus excluded from the main analysis (*N*=1,105) is higher than any count of missingness for any single criterion.
CUD: cannabis use disorder; DSM-5: Diagnostic and Statistical Manual of Mental Disorders, Fifth Edition.

**Supplementary Table 2.** Estimated percentages and relative odds of missing DSM-5 CUD identification among people who report past-year cannabis use

| Sociodemographic variable | Missing DSM-5 CUD identification | | |
| --- | --- | --- | --- |
|  | Row Wt. % (95% CI) | OR (95% CI) | aOR (95% CI) |
| Age |  |  |  |
| 12–17 years old | 7.1 (5.7, 8.8) | **5.08 (3.60, 7.16)** | **4.61 (3.27, 6.50)** |
| 18–25 years old | 2.4 (1.9, 2.9) | **1.60 (1.19, 2.17)** | **1.50 (1.11, 2.04)** |
| 26–34 years old | 1.6 (1.2, 2.1) | 1.05 (0.72, 1.55) | 1.02 (0.69, 1.51) |
| 35–49 years old | 1.5 (1.2, 1.9) | 1 [Reference] | 1 [Reference] |
| 50–64 years old | 1.6 (1.1, 2.4) | 1.09 (0.71, 1.67) | 1.11 (0.73, 1.68) |
| ≥65 years old | 1.7 (1.0, 3.1) | 1.18 (0.63, 2.20) | 1.24 (0.65, 2.38) |
| Race/ethnicity |  |  |  |
| Hispanic | 2.4 (1.8, 3.1) | **1.45 (1.05, 2.01)** | **1.42 (1.02, 1.96)** |
| Multiracial | 2.6 (1.8, 3.6) | **1.56 (1.05, 2.33)** | **1.47 (1.00, 2.15)** |
| NH American Indian or Alaska Native | 3.6 (1.9, 6.7) | **2.24 (1.19, 4.23)** | 1.71 (0.87, 3.37) |
| NH Asian, Native Hawaiian, or Pacific Islander | 3.2 (1.5, 6.6) | 1.94 (0.90, 4.19) | 1.97 (0.93, 4.17) |
| NH Black or African American | 2.7 (2.2, 3.5) | **1.67 (1.23, 2.27)** | **1.76 (1.33, 2.34)** |
| NH White | 1.7 (1.4, 2.0) | 1 [Reference] | 1 [Reference] |
| Sex |  |  |  |
| Male | 1.9 (1.6, 2.2) | 1 [Reference] | 1 [Reference] |
| Female | 2.2 (1.8, 2.7) | 1.19 (0.92, 1.53) | 1.11 (0.85, 1.46) |
| Insurance status |  |  |  |
| Private only | 2.0 (1.6, 2.4) | 1 [Reference] | 1 [Reference] |
| Any public | 2.2 (1.8, 2.6) | 1.11 (0.87, 1.42) | 0.96 (0.73, 1.25) |
| Other insurance | 2.2 (1.3, 3.7) | 1.10 (0.65, 1.88) | 1.02 (0.58, 1.82) |
| Uninsured | 1.6 (1.2, 2.2) | 0.81 (0.54, 1.20) | 0.78 (0.52, 1.17) |
| Annual income |  |  |  |
| <$20,000 | 2.1 (1.8, 2.5) | 1 [Reference] | 1 [Reference] |
| $20,000–49,999 | 2.0 (1.6, 2.6) | 0.96 (0.73, 1.26) | 0.99 (0.76, 1.29) |
| $50,000–74,999 | 2.2 (1.6, 3.0) | 1.05 (0.75, 1.47) | 1.15 (0.80, 1.65) |
| ≥$75,000 | 1.9 (1.5, 2.3) | 0.87 (0.68, 1.12) | 1.02 (0.78, 1.33) |
| Residence |  |  |  |
| Large metropolitan area | 1.9 (1.6, 2.3) | 1 [Reference] | 1 [Reference] |
| Small metropolitan area | 1.9 (1.6, 2.2) | 0.99 (0.79, 1.24) | 1.03 (0.84, 1.26) |
| Nonmetropolitan area | 2.8 (2.0, 3.9) | **1.48 (1.00, 2.18)** | **1.65 (1.16, 2.35)** |
| Survey year |  |  |  |
| 2021 | 3.4 (2.8, 4.0) | 1 [Reference] | 1 [Reference] |
| 2022 | 1.5 (1.1, 1.9) | **0.43 (0.32, 0.57)** | **0.43 (0.33, 0.58)** |
| 2023 | 1.4 (1.1, 1.7) | **0.40 (0.31, 0.51)** | **0.41 (0.32, 0.52)** |

*Notes*. Bolded values indicate 95% CI does not include 1. Data from the 2021–2023 National Survey on Drug Use and Health. Unweighted total sample of *N*=41,078. Weighted *N*=59,0157,510.

CUD: cannabis use disorder; DSM-5: Diagnostic and Statistical Manual of Mental Disorders, Fifth Edition; NH: non-Hispanic.

**Supplementary Table 3.** Crosswalk comparing the determination of meeting each DSM-5 criteria for cannabis use disorder using the DSM-5-proxy measures before and after 2020 and the DSM-5 measure after 2020 using items from the National Survey on Drug Use and Health

| **DSM-5 criteria for CUD and description** | **Variable names, item text, and logic to determine whether criteria is met** | | |
| --- | --- | --- | --- |
|  | **DSM-5-proxy measure before 2020** | **DSM-5-proxy measure after 2020** | **DSM-5 measure after 2020** |
| **1. Limits**  Cannabis is often taken in larger amounts or over a longer period than was intended | **Variable names and item text**  MRJLIMIT  During the past 12 months, did you try to set limits on how often or how much marijuana or hashish you would use?  MRJKPLMT  Were you able to keep to the limits you set, or did you often use marijuana or hashish more than you intended to?  **Logic**  YES IF (MRJLIMIT YES AND MRJKPLMT YES) | **Variable names and item text**  UDMJLRGAMTS  During the past 12 months, were there many times when you ended up using marijuana or any cannabis product in larger amounts or for a longer  time than you meant to?  **Logic**  YES IF (UDMJLRGAMTS YES), NO IF ELSE  MISSING IF (UDMJLRGAMTS 85, 94, 97, 98) | **Variable names and item text**  UDMJLRGAMTS  During the past 12 months, were there many times when you ended up using marijuana or any cannabis product in larger amounts or for a longer  time than you meant to?  **Logic**  YES IF (UDMJLRGAMTS YES), NO IF ELSE  MISSING IF (UDMJLRGAMTS 85, 94, 97, 98) |
| **2. Cutting down**  There is a persistent desire or unsuccessful efforts to cut down or control cannabis use | **Variable names and item text**  MRJCUTDN  During the past 12 months, did you want to or try to cut down or stop using marijuana or hashish?  MRJCUTEV  During the past 12 months, were you able to cut down or stop using marijuana or hashish every time you wanted to or tried to?  **Logic**  YES IF (MRJCUTDN YES AND MRJCUTEV NO) | **Variable names and item text**  UDMJTRYSTOP  During the past 12 months, did you try to cut down or try to stop using marijuana or any cannabis product?  UDMJNOTSTOP  Some people who use marijuana or any cannabis product try to cut down or stop but find they can’t. Was there more than one time in the past 12 months when you tried but were unable to cut down or stop using marijuana or any cannabis product?  UDMJWSHSTOP  In the past 12 months, did you often wish that you could cut down or stop using marijuana or any cannabis product?  **Logic**  YES IF ((UDMJTRYSTOP YES AND UDMJNOTSTOP YES) OR (UDMJWSHSTOP YES)),  NO IF ELSE  MISSING IF ((UDMJTRYSTOP 85, 94, 97, 98 OR UDMJNOTSTOP 85, 94, 97, 98) AND (UDMJWSHSTOP 85, 94, 97, 98)) | **Variable names and item text**  UDMJTRYSTOP  During the past 12 months, did you try to cut down or try to stop using marijuana or any cannabis product?  UDMJNOTSTOP  Some people who use marijuana or any cannabis product try to cut down or stop but find they can’t. Was there more than one time in the past 12 months when you tried but were unable to cut down or stop using marijuana or any cannabis product?  UDMJWSHSTOP  In the past 12 months, did you often wish that you could cut down or stop using marijuana or any cannabis product?  **Logic**  YES IF ((UDMJTRYSTOP YES AND UDMJNOTSTOP YES) OR (UDMJWSHSTOP YES)),  NO IF ELSE  MISSING IF ((UDMJTRYSTOP 85, 94, 97, 98 OR UDMJNOTSTOP 85, 94, 97, 98) AND (UDMJWSHSTOP 85, 94, 97, 98)) |
| **3. Spending time getting cannabis**  A great deal of time is spent in activities necessary to obtain cannabis, use cannabis, or recover from its effects | **Variable names and item text**  MRJLOTTM During the past 12 months, was there a month or more when you spent a lot of your time getting or using marijuana or hashish?  MRJGTOVR  During the past 12 months, was there a month or more when you spent a lot of your time getting over the effects of the marijuana or hashish you used?  **Logic**  YES IF (MRJLOTTM YES OR MRJGTOVR YES) | **Variable names and item text**  UDMJTIMEUSE  During the past 12 months, did you spend a great deal of your time using marijuana or any cannabis product, feeling its effects, or getting over the effects of marijuana or any cannabis product?  UDMJTIMEGET  During the past 12 months, did you spend a great deal of your time getting or trying to get marijuana or any cannabis product?  **Logic**  YES IF (UDMJTIMEUSE YES OR UDMJTIMEGET YES),  NO IF ELSE  MISSING IF ((UDMJTIMEUSE 85, 94, 97, 98) AND (UDMJTIMEGET 85, 94, 97, 98)) | **Variable names and item text**  UDMJTIMEUSE  During the past 12 months, did you spend a great deal of your time using marijuana or any cannabis product, feeling its effects, or getting over the effects of marijuana or any cannabis product?  UDMJTIMEGET  During the past 12 months, did you spend a great deal of your time getting or trying to get marijuana or any cannabis product?  **Logic**  YES IF (UDMJTIMEUSE YES OR UDMJTIMEGET YES),  NO IF ELSE  MISSING IF ((UDMJTIMEUSE 85, 94, 97, 98) AND (UDMJTIMEGET 85, 94, 97, 98)) |
| **4. Craving**  Craving, or a strong desire or urge to use cannabis | Not included in DSM-5-proxy measure | Not included in DSM-5-proxy measure | **Variable names and item text**  UDMJWANTBAD  During the past 12 months, were there times when you wanted to use marijuana or any cannabis product so badly that you couldn't think of anything  else?  UDMJSTRURGE  During the past 12 months, were there times when you had a strong urge to use marijuana or any cannabis product?  **Logic**  YES IF (UDMJWANTBAD YES OR UDMJSTRURGE YES),  NO IF ELSE  MISSING IF ((UDMJWANTBAD 85, 94, 97, 98) AND (UDMJSTRURGE 85, 94, 97, 98)) |
| **5. Problems at home, work, or school**  Recurrent cannabis use resulting in a failure to fulfil major role obligations at work, school, or home | **Variable names and item text**  MRJSERPB  Sometimes people who use marijuana or hashish have serious problems at home, work or school — such as: neglecting their children, missing work or school, doing a poor job at work or school, losing a job or dropping out of school. During the past 12 months, did using marijuana or hashish cause you to have serious problems like this either at home, work, or school?  **Logic**  YES IF (MRJSERPB YES) | **Variable names and item text**  UDMJWORKPRB  Sometimes people who use marijuana or any cannabis product have serious problems at work, school, or home—such as: missing a lot of work or school; getting demoted, having your hours cut, or losing a job; not being able to get a job or keep a job; getting suspended, expelled, or dropping out of school; failing to take care of family. During the past 12 months, did you have any serious problems like these at work, school, or home because of your use of marijuana or any cannabis  product?  **Logic**  YES IF (UDMJWORKPRB YES),  NO IF ELSE  MISSING IF (UDMJWORKPRB 85, 94, 97, 98) | **Variable names and item text**  UDMJWORKPRB  Sometimes people who use marijuana or any cannabis product have serious problems at work, school, or home—such as: missing a lot of work or school; getting demoted, having your hours cut, or losing a job; not being able to get a job or keep a job; getting suspended, expelled, or dropping out of school; failing to take care of family. During the past 12 months, did you have any serious problems like these at work, school, or home because of your use of marijuana or any cannabis  product?  **Logic**  YES IF (UDMJWORKPRB YES),  NO IF ELSE  MISSING IF (UDMJWORKPRB 85, 94, 97, 98) |
| **6. Problems with friends and family**  Continued cannabis use despite having persistent or recurrent social or interpersonal problems caused or exacerbated by the effects of cannabis | **Variable names and item text**  MRJFMFPB  During the past 12 months, did you have any problems with family or friends that were probably caused by your use of marijuana or hashish?  MRJFMCTD  Did you continue to use marijuana or hashish even though you thought it caused problems with family or friends?  **Logic**  YES IF (MRJFMFPB YES AND MRJFMCTD YES) | **Variable names and item text**  UDMJFMLYPRB  During the past 12 months, did you often have arguments or other problems with family or friends that were caused or made worse by your use of  marijuana or any cannabis product?)  UDMJFMLYCTD  Did you continue to use marijuana or any cannabis product even though it often caused arguments or problems with family or friends?  **Logic^a^**  YES IF (UDMJFMLYPRB YES AND UDMJFMLYCTD YES),  NO IF ELSE  MISSING IF (UDMJFMLYPRB 85, 94, 97, 98 OR UDMJFMLYCTD 85, 94, 97, 98) | **Variable names and item text**  UDMJFMLYPRB  During the past 12 months, did you often have arguments or other problems with family or friends that were caused or made worse by your use of  marijuana or any cannabis product?)  UDMJFMLYCTD  Did you continue to use marijuana or any cannabis product even though it often caused arguments or problems with family or friends?  **Logic^a^**  YES IF (UDMJFMLYPRB YES AND UDMJFMLYCTD YES),  NO IF ELSE  MISSING IF (UDMJFMLYPRB 85, 94, 97, 98 OR UDMJFMLYCTD 85, 94, 97, 98) |
| **7. Activities**  Important social, occupational, or recreational activities are given up or reduced because of cannabis use | **Variable names and item text**  MRSLSACT  This question is about important activities such as working, going to school, taking care of children, doing fun things such as hobbies and sports, and spending time with friends and family. During the past 12 months, did using marijuana or hashish cause you to give up or spend less time doing these types of important activities?  **Logic**  YES IF (MRSLSACT YES) | **Variable names and item text**  UDMJSTOPACT  During the past 12 months, did you give up or spend a lot less time doing any of these types of important activities because of your use of marijuana  or any cannabis product?  **Logic**  YES IF (UDMJSTOPACT YES),  NO IF ELSE  MISSING IF (UDMJSTOPACT 85, 94, 97, 98) | **Variable names and item text**  UDMJSTOPACT  During the past 12 months, did you give up or spend a lot less time doing any of these types of important activities because of your use of marijuana  or any cannabis product?  **Logic**  YES IF (UDMJSTOPACT YES),  NO IF ELSE  MISSING IF (UDMJSTOPACT 85, 94, 97, 98) |
| **8. Physical danger**  Recurrent cannabis use in situations in which it is physically hazardous | **Variable names and item text**  MRJPDANG  During the past 12 months, did you regularly use marijuana or hashish and then do something where using marijuana or hashish might have put you in physical danger?  **Logic**  YES IF (MRJPDANG YES) | **Variable names and item text**  UDMJGETHURT  During the past 12 months, did you repeatedly get into situations where using marijuana or any cannabis product increased your chances of getting  physically hurt?  **Logic**  YES IF (UDMJGETHURT YES),  NO IF ELSE  MISSING IF (UDMJGETHURT  85, 94, 97, 98) | **Variable names and item text**  UDMJGETHURT  During the past 12 months, did you repeatedly get into situations where using marijuana or any cannabis product increased your chances of getting  physically hurt?  **Logic**  YES IF (UDMJGETHURT YES),  NO IF ELSE  MISSING IF (UDMJGETHURT  85, 94, 97, 98) |
| **9. Mental health problems**  Cannabis use is continued despite knowledge of having a persistent or recurrent physical or psychological problem that is likely to have been caused or exacerbated by cannabis | **Variable names and item text**  MRJEMPOB  During the past 12 months, did you have any problems with your emotions, nerves, or mental health that were probably caused or made worse by your use of marijuana or hashish?  MRJEMCTD  Did you continue to use marijuana or hashish even though you thought it was causing you to have problems with your emotions, nerves, or mental health?  MRJPHLPB  During the past 12 months, did you have any physical health problems that were probably caused or made worse by your use of marijuana or hashish?  MRJPHCTD  Did you continue to use marijuana or hashish even though you thought it was causing you to have physical problems? (Yes, MRJPHCTD = 1)  **Logic**  YES IF ((MRJEMPOB YES AND MRJEMCTD YES) OR (MRJPHLPB YES AND MRJPHCTD YES) | **Variable names and item text**  UDMJMNTLPRB  During the past 12 months, did you have any long-lasting or repeated problems with emotions or mental health that were caused or made worse by  using marijuana or any cannabis product?  UDMJMNTLCTD  Did you continue to use marijuana or any cannabis product even though it was causing long-lasting or repeated problems with your emotions or  mental health or making your emotions or mental health worse?  UDMJHLTHPRB  During the past 12 months, did you have any long-lasting or repeated physical health problems that were caused or made worse by using marijuana  or any cannabis product?  UDMJHLTHCTD  Did you continue to use marijuana or any cannabis product even though it was causing long-lasting or repeated physical health problems or making your physical health problems worse?  **Logic^a^**  YES IF ((UDMJMNTLPRB YES AND UDMJMNTLCTD YES) OR (UDMJHLTHPRB YES AND UDMJHLTHCTD YES),  NO IF ELSE  MISSING IF ((UDMJMNTLPRB 85, 94, 97, 98 OR UDMJMNTLCTD 85, 94, 97, 98) AND (UDMJHLTHPRB 85, 94, 97, 98 OR UDMJHLTHCTD 85, 94, 97, 98) | **Variable names and item text**  UDMJMNTLPRB  During the past 12 months, did you have any long-lasting or repeated problems with emotions or mental health that were caused or made worse by  using marijuana or any cannabis product?  UDMJMNTLCTD  Did you continue to use marijuana or any cannabis product even though it was causing long-lasting or repeated problems with your emotions or  mental health or making your emotions or mental health worse?  UDMJHLTHPRB  During the past 12 months, did you have any long-lasting or repeated physical health problems that were caused or made worse by using marijuana  or any cannabis product?  UDMJHLTHCTD  Did you continue to use marijuana or any cannabis product even though it was causing long-lasting or repeated physical health problems or making your physical health problems worse?  **Logic^a^**  YES IF ((UDMJMNTLPRB YES AND UDMJMNTLCTD YES) OR (UDMJHLTHPRB YES AND UDMJHLTHCTD YES),  NO IF ELSE  MISSING IF ((UDMJMNTLPRB 85, 94, 97, 98 OR UDMJMNTLCTD 85, 94, 97, 98) AND (UDMJHLTHPRB 85, 94, 97, 98 OR UDMJHLTHCTD 85, 94, 97, 98) |
| **10. Tolerance**  Tolerance, as defined by either of the following:   a. A need for markedly increased amounts of cannabis to achieve intoxication or desired effect. b. A markedly diminished effect with continued use of the same amount of cannabis | **Variable names and item text**  MRJNDMOR  During the past 12 months, did you need to use more marijuana or hashish than you used to in order to get the effect you wanted? (Yes, MRJNDMOR = 1)  MRJLSEFX  During the past 12 months, did you notice that using the same amount of marijuana or hashish had less effect on you than it used to? (Yes, MRJLSEFX = 1)  **Logic**  YES IF (MRJNDMOR YES OR MRJLSEFX YES) | **Variable names and item text**  UDMJNEEDMOR  Do you need to use a lot more marijuana or any cannabis product than you used to in order to get the feeling you want?  UDMJLESSEFF  Does using the same amount of marijuana or any cannabis product have much less effect on you than it used to?  **Logic**  YES IF (UDMJNEEDMOR YES OR UDMJLESSEFF YES),  NO IF ELSE  MISSING IF ((UDMJNEEDMOR 85, 94, 97, 98) AND (UDMJLESSEFF 85, 94, 97, 98)) | **Variable names and item text**  UDMJNEEDMOR  Do you need to use a lot more marijuana or any cannabis product than you used to in order to get the feeling you want?  UDMJLESSEFF  Does using the same amount of marijuana or any cannabis product have much less effect on you than it used to?  **Logic**  YES IF (UDMJNEEDMOR YES OR UDMJLESSEFF YES),  NO IF ELSE  MISSING IF ((UDMJNEEDMOR 85, 94, 97, 98) AND (UDMJLESSEFF 85, 94, 97, 98)) |
| **11. Withdrawal**  Withdrawal, as manifested by either of the following:   a. The characteristic withdrawal syndrome for cannabis (refer to DSM-5 for further details). b. Cannabis (or a closely related substance) is taken to relieve or avoid withdrawal symptoms | Not included in DSM-5-proxy measure | Not included in DSM-5-proxy measure | **Variable names and item text**  People may experience withdrawal symptoms when they use less or stop using marijuana or any cannabis product. During the past 12 months, did you have the following withdrawal symptoms after you used less or stopped using marijuana or any cannabis product for a while?  UDMJWDANGRY  ... Feeling irritable or angry  UDMJWDFLANX  … Feeling anxious or nervous  UDMJWDSLEEP  … Having trouble sleeping  UDMJWDAPPET  … Losing your appetite or losing weight without trying to  UDMJWDSITST  … Feeling like you couldn’t sit still  UDMJWDDEPRS  … Feeling depressed  UDMJWDSTMCH  … Stomach ache  UDMJWDSHAKE  … Shaking or tremors  UDMJWDSWEAT  … Sweating  UDMJWDFEVER  … Fever  UDMJWDCHILL  … Chills  UDMJWDHEDAC  … Headache  UDMJAVOIDWD  During the past 12 months, did you use marijuana, any cannabis product, or another drug to get over or avoid having marijuana or any cannabis  product withdrawal symptoms?  **Logic^b^**  YES IF (UDMJAVOIDWD YES, 3^c^ OR (THREE OR MORE OUT OF: UDMJWDANGRY YES OR UDMJWDFLANX YES OR UDMJWDSLEEP YES OR UDMJWDAPPET YES OR UDMJWDSITST YES OR UDMJWDDEPRS YES OR (UDMJWDSTMCH YES OR UDMJWDSHAKE YES OR UDMJWDSWEAT YES OR UDMJWDFEVER YES OR UDMJWDCHILL YES OR UDMJWDHEDAC YES)),  NO IF ELSE  MISSING IF (UDMJAVWMARJ 85, 94, 97, 98, 99 AND (((ZERO OUT OF: UDMJWDANGRY YES OR UDMJWDFLANX YES OR UDMJWDSLEEP YES OR UDMJWDAPPET YES OR UDMJWDSITST YES OR UDMJWDDEPRS YES OR (UDMJWDSTMCH YES OR UDMJWDSHAKE YES OR UDMJWDSWEAT YES OR UDMJWDFEVER YES OR UDMJWDCHILL YES OR UDMJWDHEDAC YES)) AND (THREE OR MORE OUT OF: UDMJWDANGRY 85, 94, 97, 98 OR UDMJWDFLANX 85, 94, 97, 98 OR UDMJWDSLEEP 85, 94, 97, 98 OR UDMJWDAPPET 85, 94, 97, 98 OR UDMJWDSITST 85, 94, 97, 98 OR UDMJWDDEPRS 85, 94, 97, 98 OR (UDMJWDSTMCH 85, 94, 97, 98 AND UDMJWDSHAKE 85, 94, 97, 98 AND UDMJWDSWEAT 85, 94, 97, 98 AND UDMJWDFEVER 85, 94, 97, 98 AND UDMJWDCHILL 85, 94, 97, 98 AND UDMJWDHEDAC 85, 94, 97, 98)) OR ((ONE OUT OF: UDMJWDANGRY YES OR UDMJWDFLANX YES OR UDMJWDSLEEP YES OR UDMJWDAPPET YES OR UDMJWDSITST YES OR UDMJWDDEPRS YES OR (UDMJWDSTMCH YES OR UDMJWDSHAKE YES OR UDMJWDSWEAT YES OR UDMJWDFEVER YES OR UDMJWDCHILL YES OR UDMJWDHEDAC YES)) AND (TWO OR MORE OUT OF: UDMJWDANGRY 85, 94, 97, 98 OR UDMJWDFLANX 85, 94, 97, 98 OR UDMJWDSLEEP 85, 94, 97, 98 OR UDMJWDAPPET 85, 94, 97, 98 OR UDMJWDSITST 85, 94, 97, 98 OR UDMJWDDEPRS 85, 94, 97, 98 OR (UDMJWDSTMCH 85, 94, 97, 98 AND UDMJWDSHAKE 85, 94, 97, 98 AND UDMJWDSWEAT 85, 94, 97, 98 AND UDMJWDFEVER 85, 94, 97, 98 AND UDMJWDCHILL 85, 94, 97, 98 AND UDMJWDHEDAC 85, 94, 97, 98)) OR ((TWO OUT OF: UDMJWDANGRY YES OR UDMJWDFLANX YES OR UDMJWDSLEEP YES OR UDMJWDAPPET YES OR UDMJWDSITST YES OR UDMJWDDEPRS YES OR (UDMJWDSTMCH YES OR UDMJWDSHAKE YES OR UDMJWDSWEAT YES OR UDMJWDFEVER YES OR UDMJWDCHILL YES OR UDMJWDHEDAC YES)) AND (ONE OR MORE OUT OF: UDMJWDANGRY 85, 94, 97, 98 OR UDMJWDFLANX 85, 94, 97, 98 OR UDMJWDSLEEP 85, 94, 97, 98 OR UDMJWDAPPET 85, 94, 97, 98 OR UDMJWDSITST 85, 94, 97, 98 OR UDMJWDDEPRS 85, 94, 97, 98 OR (UDMJWDSTMCH 85, 94, 97, 98 AND UDMJWDSHAKE 85, 94, 97, 98 AND UDMJWDSWEAT 85, 94, 97, 98 AND UDMJWDFEVER 85, 94, 97, 98 AND UDMJWDCHILL 85, 94, 97, 98 AND UDMJWDHEDAC 85, 94, 97, 98)))) |

85 = Bad data logically assigned; 91 = Never used marijuana; 94 = Don't know; 97 = Refused; 98 = Blank (no answer).
^a^ Based on guidance received from the Center for Behavioral Health Statistics and Quality Request Team on Criterion 6, meeting a criterion that requires both of two items’ yes conditions (i.e., yes joined by AND) requires both items to not be missing; thus, either variable in a pair being missing would make that criteria missing.
^b^ Based on guidance received from the Center for Behavioral Health Statistics and Quality Request Team on Criterion 11, missingness for this criterion is determined as a function of the number of items equal to "yes" and number of items missing as indicated here; furthermore, all ‘physical health symptom’ variables (i.e., UDMJWDSTMCH, UDMJWDSHAKE, UDMJWDSWEAT, UDMJWDFEVER, UDMJWDCHILL, and UDMJWDHEDAC) are, for purposes of meeting criteria, considered to make up one item; in other words, this criterion is missing if the UDMJAVWMARJ item is missing and either: zero items are yes and three or more items are missing; one item is yes and two or more items are missing; or two items are yes and one or more items are missing.
^c^ One individual in the 2023 public use data file used to create this table had response of “3.”

**Supplemental Table 4.** Correlates of having additionally identified cannabis use disorder and cannabis use disorder severity using the full DSM-5 measure among people who report past-year cannabis use (Model 1), and adjusting for near-daily or daily cannabis use frequency (Model 2)

| Sociodemographic variable | Additionally identified mild CUD using the full DSM-5 measure^a^ vs. no CUD using both measures | | Additionally identified moderate CUD using the full DSM-5 measure vs. mild CUD using both measures | | Additionally identified severe CUD using the full DSM-5 measure vs. moderate CUD using both measures | |
| --- | --- | --- | --- | --- | --- | --- |
|  | Model 1  aRRR^b^ (95% CI) | Model 2  aRRR^b^ (95% CI) | Model 1  aRRR^c^ (95% CI) | Model 2  aRRR^c^ (95% CI) | Model 1  aRRR^c^ (95% CI) | Model 2  aRRR^c^ (95% CI) |
| Age |  |  |  |  |  |  |
| 12–17 years old | 1.15 (0.92, 1.45) | **1.65 (1.31, 2.09)** | 1.39 (1.00, 1.93) | **1.55 (1.11, 2.15)** | 1.39 (0.76, 2.53) | 1.65 (0.88, 3.09) |
| 18–25 years old | 1.11 (0.91, 1.34) | **1.31 (1.07, 1.59)** | **1.52 (1.22, 1.90)** | **1.57 (1.26, 1.96)** | 1.65 (0.99, 2.75) | **1.75 (1.06, 2.91)** |
| 26–34 years old | 1.16 (0.97, 1.39) | **1.22 (1.02, 1.47)** | **1.44 (1.17, 1.76)** | **1.44 (1.17, 1.76)** | 1.71 (0.96, 3.04) | 1.74 (0.98, 3.09) |
| 35–49 years old | 1 [Reference] | 1 [Reference] | 1 [Reference] | 1 [Reference] | 1 [Reference] | 1 [Reference] |
| 50–64 years old | 0.80 (0.59, 1.09) | 0.80 (0.58, 1.10) | **0.55 (0.32, 0.95)** | **0.55 (0.31, 0.95)** | 1.01 (0.37, 2.77) | 1.01 (0.37, 2.77) |
| ≥65 years old | **0.20 (0.14, 0.28)** | **0.21 (0.15, 0.29)** | 0.42 (0.13, 1.34) | 0.43 (0.14, 1.34) | **0.21 (0.05, 0.89)** | **0.22 (0.05, 0.92)** |
| Race/ethnicity |  |  |  |  |  |  |
| Hispanic | **0.81 (0.67, 0.98)** | 0.89 (0.73, 1.09) | 0.87 (0.68, 1.13) | 0.92 (0.41, 1.56) | 0.87 (0.53, 1.41) | 0.92 (0.57, 1.50) |
| Multiracial | **1.45 (1.07, 1.96)** | **1.44 (1.08, 1.92)** | 1.29 (0.85, 1.96) | 1.30 (0.85, 1.98) | 0.70 (0.32, 1.52) | 0.71 (0.32, 1.54) |
| NH American Indian or  Alaska Native | 1.09 (0.60, 1.96) | 1.01 (0.56, 1.84) | 1.03 (0.69, 1.52) | 1.05 (0.71, 1.57) | **2.76 (1.06, 7.21)** | **2.85 (1.10, 7.41)** |
| NH Asian, Native Hawaiian,  or Pacific Islander | **0.53 (0.33, 0.83)** | **0.61 (0.39, 0.95)** | 0.74 (0.37, 1.46) | 0.80 (0.41, 1.56) | 1.18 (0.58, 2.43) | 1.28 (0.63, 2.62) |
| NH Black or African  American | 1.02 (0.84, 1.23) | 1.10 (0.91, 1.33) | 0.89 (0.71, 1.13) | 0.90 (0.71, 1.14) | 0.84 (0.48, 1.50) | 0.86 (0.48, 1.53) |
| NH White | 1 [Reference] | 1 [Reference] | 1 [Reference] | 1 [Reference] | 1 [Reference] | 1 [Reference] |
| Sex |  |  |  |  |  |  |
| Male | 1 [Reference] | 1 [Reference] | 1 [Reference] | 1 [Reference] | 1 [Reference] | 1 [Reference] |
| Female | **0.80 (0.71, 0.89)** | **0.84 (0.75, 0.95)** | 1.04 (0.85, 1.27) | 1.05 (0.85, 1.28) | 1.34 (0.94, 1.91) | 1.34 (0.94, 1.91) |
| Insurance status |  |  |  |  |  |  |
| Private only | 1 [Reference] | 1 [Reference] | 1 [Reference] | 1 [Reference] | 1 [Reference] | 1 [Reference] |
| Any public | **1.58 (1.35, 1.85)** | **1.51 (1.30, 1.76)** | 1.34 (0.98, 1.82) | 1.30 (0.96, 1.77) | **1.61 (1.05, 2.48)** | **1.56 (1.01, 2.41)** |
| Other insurance | 1.23 (0.81, 1.87) | 1.08 (0.69, 1.68) | 1.23 (0.78, 1.93) | 1.25 (0.79, 1.97) | 1.01 (0.39, 2.61) | 0.96 (0.37, 2.50) |
| Uninsured | **1.32 (1.10, 1.58)** | 1.21 (1.00, 1.47) | 1.23 (0.90, 1.67) | 1.20 (0.88, 1.63) | 1.42 (0.80, 2.52) | 1.36 (0.77, 2.41) |
| Annual income |  |  |  |  |  |  |
| <$20,000 | 1 [Reference] | 1 [Reference] | 1 [Reference] | 1 [Reference] | 1 [Reference] | 1 [Reference] |
| $20,000–49,999 | 1.11 (0.92, 1.33) | 1.04 (0.85, 1.26) | 0.81 (0.63, 1.03) | 0.79 (0.63, 1.00) | 1.20 (0.75, 1.92) | 1.16 (0.72, 1.86) |
| $50,000–74,999 | 0.99 (0.80, 1.23) | 0.98 (0.79, 1.21) | 0.81 (0.61, 1.07) | 0.81 (0.62, 1.07) | 0.91 (0.50, 1.63) | 0.90 (0.50, 1.63) |
| ≥$75,000 | **0.77 (0.62, 0.96)** | 0.82 (0.65, 1.02) | 0.91 (0.70, 1.17) | 0.90 (0.71, 1.15) | 1.23 (0.76, 2.00) | 1.22 (0.76, 1.98) |
| Residence |  |  |  |  |  |  |
| Large metropolitan area | 1 [Reference] | 1 [Reference] | 1 [Reference] | 1 [Reference] | 1 [Reference] | 1 [Reference] |
| Small metropolitan area | 1.18 (0.99, 1.40) | 1.18 (0.98, 1.42) | 0.94 (0.75, 1.19) | 0.94 (0.75, 1.18) | 1.06 (0.68, 1.65) | 1.05 (0.62, 1.53) |
| Nonmetropolitan area | 1.06 (0.85, 1.33) | 1.01 (0.80, 1.26) | 1.04 (0.77, 1.40) | 1.05 (0.78, 1.41) | 0.64 (0.35, 1.16) | 0.63 (0.35, 1.15) |
| Survey year |  |  |  |  |  |  |
| 2021 | 1 [Reference] | 1 [Reference] | 1 [Reference] | 1 [Reference] | 1 [Reference] | 1 [Reference] |
| 2022 | 1.03 (0.85, 1.24) | 1.04 (0.85, 1.26) | 1.17 (0.92, 1.47) | 1.18 (0.93, 1.48) | 0.97 (0.61, 1.52) | 0.97 (0.62, 1.53) |
| 2023 | 1.04 (0.86, 1.26) | 1.03 (0.85, 1.26) | 1.00 (0.77, 1.28) | 1.01 (0.78, 1.30) | 0.82 (0.51, 1.32) | 0.82 (0.52, 1.31) |
| Cannabis use frequency |  |  |  |  |  |  |
| Less than daily or near-daily | - | 1 [Reference] | - | 1 [Reference] | - | 1 [Reference] |
| Daily or near daily | - | **5.11 (4.34, 6.00)** | - | **1.32 (1.11, 1.58)** | - | **1.57 (1.05, 2.36)** |

^a^ All instances of additionally identified CUD using the full DSM-5 measure were identified as mild CUD; this group is equivalent to “additionally identified CUD using the full DSM-5 measure.”

^b^ From multinomial logistic models with categories of: no CUD using both measures, additionally identified CUD using the full DSM-5 measure, and CUD using both measures.

^c^ From multinomial logistic models with categories of: no CUD using both measures; additionally identified mild CUD using the full DSM-5; mild CUD using both measures; additionally identified moderate CUD using the full DSM-5 measure; moderate CUD using both measures; additionally identified severe CUD using the full DSM-5 measure; and severe CUD using both measures.

*Notes*. Bolded values indicate 95% CI does not include 1. Data from the 2021–2023 National Survey on Drug Use and Health.

CUD: cannabis use disorder; DSM-5: Diagnostic and Statistical Manual of Mental Disorders, Fifth Edition; NH: non-Hispanic.

**Supplementary Table 5.** Estimated percentage of people meeting each DSM-5 cannabis use disorder criteria and case classification using the DSM-5 and DSM-5-proxy measures among people reporting past-year cannabis use

| **DSM-5 CUD Criteria** | **Meeting criterion**  Column Wt. % (95% CI) | **Additionally identified CUD using the full DSM-5 measure**  Row Wt. % (95% CI) | **CUD using both measures**  Row Wt. % (95% CI) | **No CUD using both measures**  Row Wt. % (95% CI) |
| --- | --- | --- | --- | --- |
| Criterion 1 – Limits | 14.7 (14.0, 15.3) | 4.7 (3.7, 5.9) | 91.2 (89.7, 92.4) | 4.2 (3.4, 5.1) |
| Criterion 2 – Cutting down | 8.8 (8.2, 9.3) | 5.0 (3.7, 6.8) | 86.2 (83.3, 88.7) | 8.8 (7.0, 11.1) |
| Criterion 3 – Spending time | 29.7 (28.9, 30.4) | 14.8 (13.8, 15.9) | 68.1 (66.5, 69.7) | 17.1 (15.7, 18.6) |
| Criterion 4 – Craving | 28.6 (27.8, 29.4) | 25.0 (23.5, 26.5) | 58.8 (57.4, 60.3) | 16.2 (15.1, 17.4) |
| Criterion 5 – Problems at home, work, or school | 2.4 (2.1, 2.6) | 1.3 (0.7, 2.5) | 97.0 (95.1, 98.2) | 1.7 (0.9, 3.0) |
| Criterion 6 – Problems with friends and family | 2.2 (2.0, 2.4) | 2.7 (1.2, 6.1) | 96.2 (92.2, 98.2) | 1.1 (0.4, 2.7) |
| Criterion 7 – Activities | 4.6 (4.2, 4.9) | 1.6 (1.0. 2.7) | 95.4 (93.2, 96.8) | 3.0 (1.7, 5.3) |
| Criterion 8 – Physical danger | 1.2 (1.0, 1.4) | 4.0 (1.9, 8.3) | 91.5 (84.1, 95.7) | 4.5 (1.6, 12.1) |
| Criterion 9 – Mental health problems | 3.9 (3.5, 4.2) | 2.2 (1.2, 4.0) | 96.2 (93.7, 97.7) | 1.6 (0.7, 3.7) |
| Criterion 10 – Tolerance | 20.1 (19.4, 20.8) | 7.4 (6.4, 8.4) | 81.9 (80.5, 83.3) | 10.7 (9.7, 11.8) |
| Criterion 11 – Withdrawal | 14.0 (13.4, 14.6) | 20.3 (18.2, 22.7) | 73.3 (71.0, 75.5) | 6.4 (5.5, 7.4) |

*Note*s. Unweighted total sample of *n* = 39,973. Data from the 2021–2023 National Survey on Drug Use and Health.

CUD: cannabis use disorder; DSM-5: Diagnostic and Statistical Manual of Mental Disorders, Fifth Edition.

**Supplemental Table 6.** Estimated percentages and correlates of meeting “craving” and “withdrawal” DSM-5 cannabis use disorder criteria among people reporting past-year cannabis use

| Sociodemographic variable | Meets “craving” criterion^a^ | |  | Meets “withdrawal” criterion^b^ | |
| --- | --- | --- | --- | --- | --- |
|  | Row Wt. % (95% CI) | RR (95% CI) |  | Row Wt. % (95% CI) | RR (95% CI) |
| Age |  |  |  |  |  |
| 12–17 years old | 37.2 (34.7, 39.8) | **1.38 (1.26, 1.52)** |  | 24.8 (22.6, 27.2) | **2.07 (1.77, 2.41)** |
| 18–25 years old | 38.2 (36.6, 39.7) | **1.42 (1.29, 1.56)** |  | 22.6 (21.4, 23.7) | **1.88 (1.70, 2.09)** |
| 26–34 years old | 33.0 (31.6, 34.4) | **1.23 (1.12, 1.34)** |  | 15.1 (13.8, 16.5) | **1.26 (1.08, 1.47)** |
| 35–49 years old | 26.9 (25.0, 28.9) | 1 [Reference] |  | 12.0 (10.8, 13.3) | 1 [Reference] |
| 50–64 years old | 18.3 (15.8, 21.0) | **0.68 (0.58, 0.80)** |  | 7.0 (5.5, 9.0) | **0.59 (0.45, 0.77)** |
| ≥65 years old | 13.9 (11.7, 16.5) | **0.52 (0.43, 0.63)** |  | 3.0 (1.9, 4.8) | **0.25 (0.15, 0.41)** |
| Race/ethnicity |  |  |  |  |  |
| Hispanic | 28.5 (26.6, 30.5) | 1.02 (0.93, 1.11) |  | 16.9 (15.5, 18.4) | **1.37 (1.24, 1.52)** |
| Multiracial | 34.3 (30.3, 38.5) | **1.22 (1.08, 1.39)** |  | 20.3 (16.8, 24.2) | **1.64 (1.37, 1.98)** |
| NH American Indian or Alaska Native | 37.8 (30.9, 45.3) | **1.35 (1.12, 1.64)** |  | 21.7 (15.7, 29.1) | **1.76 (1.30, 2.38)** |
| NH Asian, Native Hawaiian, or Pacific Islander | 24.2 (19.9, 29.2) | 0.86 (0.71, 1.05) |  | 12.5 (9.6, 16.0) | 1.01 (0.79, 1.30) |
| NH Black or African American | 30.9 (28.5, 33.4) | **1.10 (1.01, 1.21)** |  | 16.8 (15.3, 18.4) | **1.36 (1.21, 1.53)** |
| NH White | 28.0 (26.8, 29.2) | 1 [Reference] |  | 12.3 (11.6, 13.1) | 1 [Reference] |
| Sex |  |  |  |  |  |
| Male | 30.5 (29.3, 31.7) | 1 [Reference] |  | 14.3 (13.4, 15.3) | 1 [Reference] |
| Female | 26.4 (25.4, 27.4) | **0.87 (0.82, 0.91)** |  | 13.6 (12.8, 14.4) | 0.95 (0.86, 1.04) |
| Insurance status |  |  |  |  |  |
| Private only | 26.3 (25.2, 27.4) | 1 [Reference] |  | 11.0 (10.2, 11.9) | 1 [Reference] |
| Any public | 29.7 (28.2, 31.2) | **1.13 (1.05, 1.21)** |  | 16.3 (15.3, 17.4) | **1.48 (1.34, 1.64)** |
| Other insurance | 33.9 (29.6, 38.4) | **1.29 (1.12, 1.48)** |  | 18.5 (15.1, 22.5) | **1.68 (1.35, 2.09)** |
| Uninsured | 34.1 (31.5, 36.8) | **1.30 (1.19, 1.42)** |  | 18.1 (15.7, 20.7) | **1.64 (1.40, 1.91)** |
| Annual income |  |  |  |  |  |
| <$20,000 | 32.8 (31.1, 34.5) | 1 [Reference] |  | 18.5 (17.3, 19.8) | 1 [Reference] |
| $20,000–49,999 | 30.9 (29.3, 32.6) | 0.94 (0.88, 1.02) |  | 16.7 (15.4, 18.0) | **0.90 (0.82, 0.99)** |
| $50,000–74,999 | 30.7 (28.0, 33.6) | 0.94 (0.86, 1.02) |  | 13.2 (12.1, 14.4) | **0.71 (0.64, 0.80)** |
| ≥$75,000 | 24.4 (23.3, 25.5) | **0.74 (0.69, 0.80)** |  | 10.3 (9.4, 11.3) | **0.56 (0.50, 0.62)** |
| Residence |  |  |  |  |  |
| Large metropolitan area | 27.7 (26.5, 28.9) | 1 [Reference] |  | 13.5 (12.7, 14.4) | 1 [Reference] |
| Small metropolitan area | 29.8 (28.8, 30.9) | **1.08 (1.03, 1.13)** |  | 14.2 (13.2, 15.2) | 1.05 (0.95, 1.15) |
| Nonmetropolitan area | 29.8 (27.1, 32.6) | 1.08 (0.97, 1.20) |  | 15.5 (14.0, 17.1) | **1.15 (1.03, 1.28)** |
| Survey year |  |  |  |  |  |
| 2021 | 28.5 (26.7, 30.3) | 1 [Reference] |  | 13.4 (12.3, 14.6) | 1 [Reference] |
| 2022 | 28.8 (27.5, 30.1) | 1.01 (0.93, 1.10) |  | 14.1 (13.2, 15.2) | 1.05 (0.94, 1.18) |
| 2023 | 28.5 (27.2, 29.9) | 1.00 (0.92, 0.98) |  | 14.3 (13.4, 15.1) | 1.06 (0.96, 1.18) |
| Cannabis use frequency |  |  |  |  |  |
| Less than daily or near-daily | 19.7 (18.9, 20.6) | 1 [Reference] |  | 9.3 (8.7, 9.9) | 1 [Reference] |
| Daily or near daily | 55.3 (53.5, 57.1) | **2.81 (2.66, 2.96)** |  | 28.0 (26.4, 29.5) | **3.01 (2.77, 3.27)** |

^a^ Unweighted *N* = 39,968; five individuals who had sufficient information to determine DSM-5 CUD classification were missing items to determine the “craving” criteria
^b^ Unweighted N = 39,916; 47 individuals who had sufficient information to determine DSM-5 CUD classification were missing items to determine the “withdrawal” criteria
*Notes*. Bolded values indicate 95% CI does not include 1. Data from the 2021–2023 National Survey on Drug Use and Health. No adjusted relative risks are provided because the full adjusted models did not converge.
CUD: cannabis use disorder; DSM-5: Diagnostic and Statistical Manual of Mental Disorders, Fifth Edition; NH: non-Hispanic.
